# Supplementary material for: Tuberculous Pleural Effusion-Derived Exosomal miR-130b-3p and miR-423-5p Promote the Proliferation of Lung Cancer Cells via Cyclin D1
Source: Int J Mol Sci. 2024 Sep 20;25(18):10119. doi: 10.3390/ijms251810119 (PMC11431986; doi:10.3390/ijms251810119)
Supplement: Supplementary file 1 [file ijms-25-10119-s001.zip › ijms-3183776-supplementary.pdf]

International Journal of
Molecular Sciences

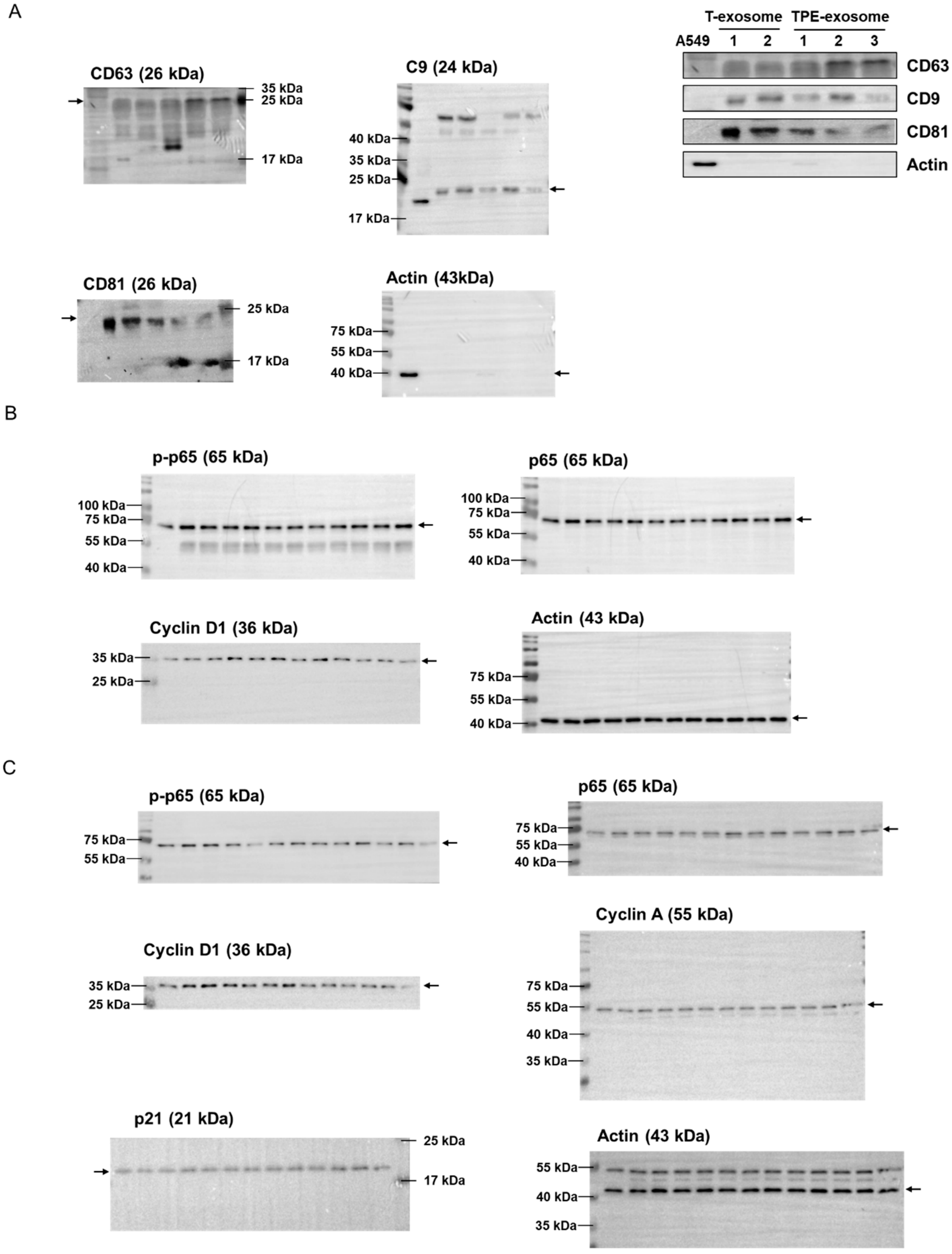

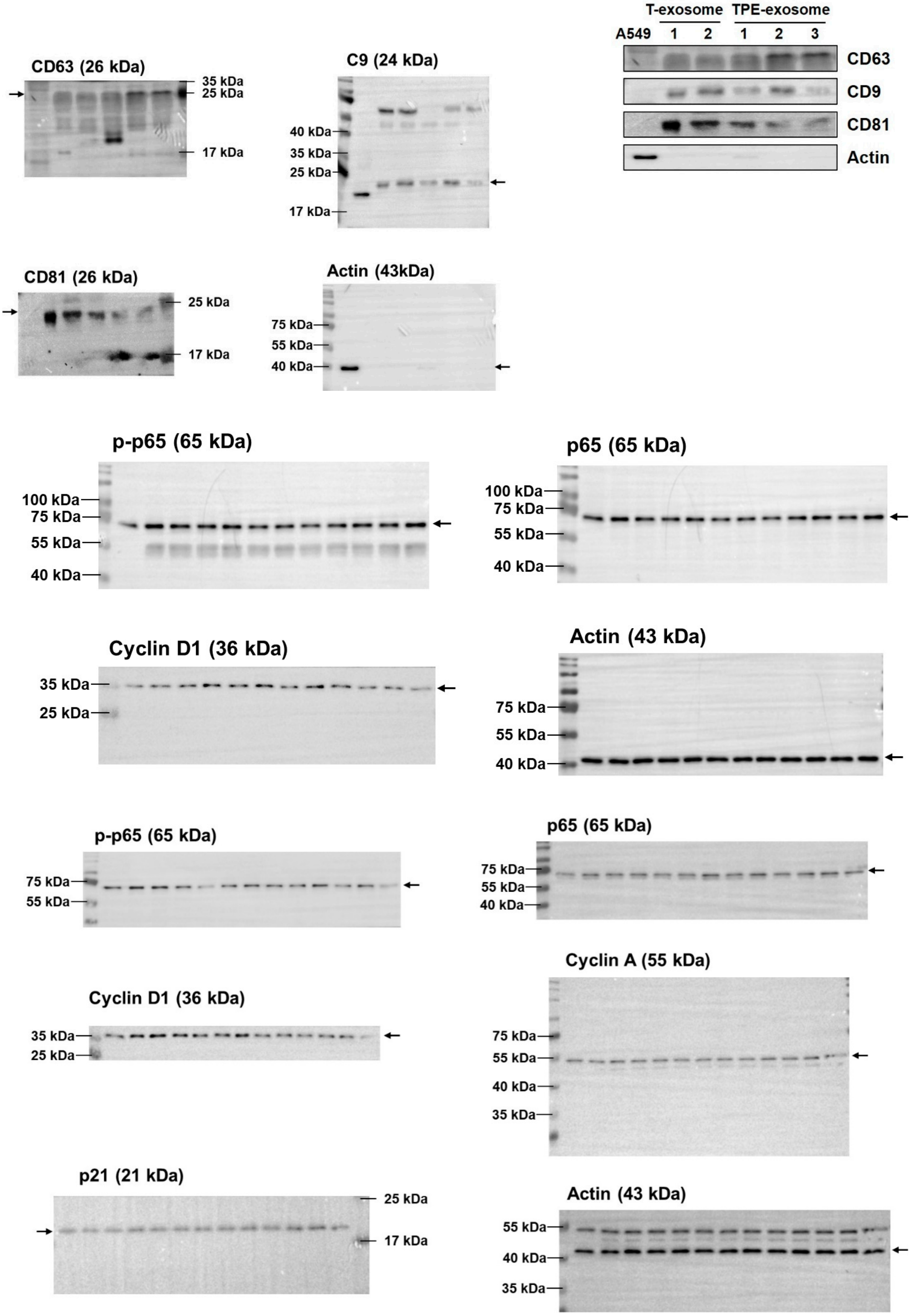

Figure S1. Original unprocessed images of Western blotting. (a) Figure 1B, (b) Figure 3C, (c) Figure 4C

Supplementary Table S1. Human sequences and assession numbers for miRNA primers used in qRT-PCR.

Gene	primer Sequences (5'-3')
hsa-miR-130b-3p	CAGTGCAATGATGAAAGGGCAT
hsa-miR-423-5p	TGAGGGGCAGAGAGCGAGACTTT
hsa-miR-320b	AAAAGCTGGGTTGAGAGGGCAA
hsa-miR-374a-5p	CGTTATAATACAACCTGATAAGTG
hsa-miR-409-3p	GAATGTTGCTCGGTGAACCCCT
